# История медикаментозного лечения ожирения

**DOI:** 10.14341/probl13469

**Published:** 2025-05-20

**Authors:** М. А. Берковская, О. Ю. Гурова, Е. В. Суркова, В. В. Фадеев

**Affiliations:** Первый Московский государственный медицинский университет имени И.М. Сеченова; Первый Московский государственный медицинский университет имени И.М. Сеченова; Первый Московский государственный медицинский университет имени И.М. Сеченова; Первый Московский государственный медицинский университет имени И.М. Сеченова

**Keywords:** ожирение, фармакотерапия ожирения, препараты для снижения массы тела

## Abstract

Эпидемия ожирения является важнейшей проблемой современного общественного здоровья и здравоохранения. Патофизиология ожирения, лежащая в основе его хронического, прогрессирующего течения, определяет трудности разработки эффективных и безопасных методов контроля массы тела. В настоящей статье представлена ​​краткая история фармакотерапии ожирения, обсуждается статус имеющихся в настоящее время препаратов для снижения массы тела, а также перспективы медикаментозного лечения ожирения.

## Abstract (en)

The obesity epidemic is a major public health problem nowadays. The pathophysiology of obesity, which underlies its chronic, progressive, self-sustaining course, determines the difficulties in developing effective and safe methods for body weight control. The article is dedicated to the consideration of the evolution of conservative treatment of obesity, in particular, the history of pharmacotherapy of this disease, characterized by many ups and downs, is presented. The paper discusses, in chronological order, the emergence, efficacy, mechanisms of action, and described side effects of drugs that were investigated and used for weight loss at one time or another, as well as the reasons why many of them were subsequently withdrawn from the market. Until recently, long-term effective and safe pharmacotherapy of obesity remained an insurmountable challenge. Only in the last two decades has the understanding of the molecular mechanisms of appetite control reached a level that allows for a more productive search and development of promising drugs aimed at the pathogenetic treatment of obesity. This article discusses the status of currently available drugs for weight loss, as well as the prospects for drug treatment of obesity. The results of clinical trials of advanced therapeutic molecules, including gastrointestinal hormone receptor agonists, reinforce the belief that a breakthrough in the drug treatment of obesity is possible.

## ВВЕДЕНИЕ

Разработка эффективных методов лечения ожирения является одной из важнейших задач современного здравоохранения. Распространенность ожирения в мире почти утроилась с 1975 года и продолжает прогрессивно нарастать. По данным ВОЗ на 2022 г., 43% взрослых в возрасте 18 лет и старше имели избыточный вес, а 16% страдали ожирением [1].

Ожирение является важным фактором риска многих коморбидных состояний, в первую очередь сахарного диабета 2 типа (СД 2), сердечно-сосудистых, а также ряда онкологических, желудочно-кишечных, скелетно-мышечных заболеваний и психологических проблем. Ожирение, особенно развившееся в возрасте до 60 лет, связано с повышением смертности на 45–61% [2]. Кроме того, чрезвычайно значимым является финансовое бремя ожирения для современных систем здравоохранения.

Понимание природы ожирения как хронического, рецидивирующего, системного заболевания фактически положило конец распространенному заблуждению о том, что оно является результатом «недостаточной самодисциплины». Центральным аргументом, определяющим ожирение как хроническое заболевание, является его особая патофизиология, которая приводит к избыточному накоплению и дисфункции жировой ткани, что в сочетании с гомеостатическими механизмами препятствует снижению массы тела и способствует ее дальнейшему увеличению [3]. Эти патофизиологические механизмы могут объяснить, почему краткосрочные поведенческие вмешательства часто недостаточны для долгосрочного удержания потери массы тела.

Поскольку модификация образа жизни обеспечивает умеренную и краткосрочную эффективность [4], усиление стратегии лечения ожирения может быть достигнуто путем применения фармакологических и/или хирургических вмешательств. Бариатрическая хирургия представляет собой наиболее эффективный подход к снижению массы тела, приводящий к снижению смертности от сердечно-сосудистых заболеваний или рака на 30% и 23% соответственно [5]. Современная бариатрическая хирургия при морбидном ожирении увеличивает общую продолжительность жизни на 3 года [5], приводит к значимому и устойчивому снижению артериального давления, уровня глюкозы и показателей липидного обмена [6]. Тем не менее хирургические вмешательства имеют свои ограничения, а также не в состоянии удовлетворить глобальные потребности в медицинской помощи людям с ожирением. В этой связи был и остается актуальным вопрос о разработке эффективных и безопасных медикаментозных методов лечения, обеспечивающих значимое и долгосрочное снижение массы тела и уменьшение рисков коморбидных состояний.

История фармакотерапии ожирения характеризуется множеством взлетов и падений. Многие препараты, которые были исследованы и применялись для снижения массы тела, впоследствии были отозваны с рынка в связи с проблемами безопасности. До недавнего времени долгосрочная эффективная и безопасная фармакотерапия ожирения оставалась непреодолимой проблемой. Только в последние два десятилетия понимание молекулярных механизмов контроля аппетита достигло уровня, позволяющего проводить более продуктивный поиск и разработку перспективных препаратов, направленных на патогенетическое лечение ожирения. Результаты клинических испытаний передовых терапевтических молекул, включая агонистов рецепторов гормонов желудочно-кишечного тракта, укрепляют веру в возможность прорыва в медикаментозном лечении ожирения.

## ОТНОШЕНИЕ К ОЖИРЕНИЮ С ДРЕВНИХ ВРЕМЕН ДО НАШИХ ДНЕЙ

Имеются данные о том, что ожирение встречалось (хотя и редко) в библейские времена (около 3500 лет назад) [7], однако понимание опасности ожирения начало осознаваться только около 500 г. до н.э. В это время индийский врач Сушрута описал болезнь «Мадху-меха», которая вызывала у пациентов «сладкий вкус и запах, как у меда». Сушрута пророчески писал, что «Мадху-меха» была связана либо с врожденным дефектом, из-за которого пациенты худели, страдали от сухости тела, чрезмерной жажды и потери аппетита, либо с «неразумной» диетой, симптомы которой включали «ожирение, прожорливость, блеск тела и повышенную сонливость» [7]. Можно предполагать, что сегодня описанные заболевания известны нам как сахарный диабет 1 и 2 типов соответственно.

Древние греки знали об ожирении как о риске для здоровья, описывая, в частности, его взаимосвязь с нерегулярным менструальным циклом и бесплодием у женщин. Гиппократ (460–377 гг. до н.э.) отмечал, что преждевременная смерть чаще встречается у полных людей, чем у худых. Он писал: «Очень вредно для здоровья принимать больше пищи, чем может выдержать телосложение, когда в то же время человек не использует упражнений, чтобы избавиться от этого избытка» [7]. Выдающийся римский врач, хирург и философ Гален (129–200 гг. н.э.) упоминал о полисаркосе (болезненно тучном) человеке, описание которого происходит от слов «поли» (много) и «сарка» (плоть). Полисаркос «не может ходить, не потея, не может дотянуться сидя за столом из-за массы желудка, не может свободно дышать, не может родить, не может очиститься» [8].

С появлением христианства в Европе выросла вера в то, что болезнь возникает из-за греха и, следовательно, является наказанием от Бога. В этот период ожирение было обычным явлением среди элиты и означало богатство, однако чревоугодие считалось одним из «семи смертных грехов» католической церкви. В отличие от Европы, исламская империя Юго-Западной и Центральной Азии в то время внесла большой вклад в развитие медицины. Авиценна, видный деятель арабской медицинской традиции, упоминал ожирение и CД2 в своей медицинской энциклопедии начала XII века, описывая «сладкий вкус диабетической мочи» [7].

К XVIII веку в Европе отношение к ожирению снова начало меняться: шотландский врач Джордж Чейн пропагандировал физические упражнения, свежий воздух, «воды для ванн» и обильное количество овощей для сдерживания ожирения. В 1800-х годах некоторые британские врачи рекомендовали диеты с низким содержанием углеводов и высоким содержанием белка для сдерживания ожирения [7].

## ИСТОРИЧЕСКИЕ МЕТОДЫ ЛЕЧЕНИЯ ОЖИРЕНИЯ

Рекомендации по лечению ожирения восходят к древнегреческим и римским временам. Врачи во времена Гиппократа предлагали людям с избыточным весом «сократить прием пищи и избегать питья до отказа», а также регулярно заниматься физическими упражнениями, особенно «бегать ночью» и «прогуливаться ранним утром» [9].

Некоторые также рекомендовали рвотные и слабительные средства — одни из первых препаратов для снижения веса. Рвотные средства, включая растения морозника и медовую воду, рекомендовали «для эвакуации пищи два или три раза в месяц всем мужчинам и женщинам». Слабительные средства готовили, например, из сока скаммонии (вьюнка), книдской ягоды и морского молочая. Мягкие слабительные включали ослиное молоко с медом, дикую петрушку, повилику тимьяновую (Cuscuta epithymum), медовую воду или сладкое вино [9]. В трудах Элиана (170–235 гг. н.э.) сообщается об использовании, возможно, самого раннего метода лечения апноэ во сне, когда врачи «приготовили очень длинные тонкие иглы и проткнули ими бедра и живот древнегреческого государственного деятеля Дионисия, когда он погрузился в глубокий сон» [10].

Гален описал методы лечения ожирения, которые включали напряженный бег каждое утро с последующей теплой ванной, легкой пищей и дополнительной физической работой [10]. Сушрута рекомендовал физическую работу и описал дополнительные средства от ожирения, такие как «голодание, упражнения и истощающие меры». В 1620-х годах британский врач Тобиас Веннер впервые использовал термин “obese” для описания людей с избыточным весом и рекомендовал купаться в теплых источниках Бата, «чтобы сделать стройными те тела, которые слишком грубы» [6].

В начале XX века люди с ожирением начали искать эффективные средства, чтобы достичь общественного идеала стройности; нередко производители наживались на их наивности, предлагая широкий спектр шарлатанских средств. Так, «препарат» Fatoff предназначался для лечения избыточного веса и ожирения, но содержал в своем составе лишь 10% мыла и 90% воды [11]. Точно так же позже было обнаружено, что продукт Human-Ease, продаваемый как лекарство от ожирения и «всех известных болезней», на 95% состоит из сала [11].

## ФАРМАКОТЕРАПИЯ ОЖИРЕНИЯ

Эра медикаментозной терапии ожирения началась в конце XIX века, когда с целью снижения массы тела начали назначать гормоны щитовидной железы, применяемые для лечения гипотиреоза [12].

Фармакотерапия ожирения имеет долгую и сложную историю, вмещающую целую череду первоначально многообещающих препаратов, впоследствии отозванных из соображений безопасности. Лекарственные препараты, которые были исследованы для лечения ожирения, включают самые разнообразные агенты, такие как митохондриальные разобщители, симпатомиметики, серотонинергические средства, ингибиторы кишечной липазы, антагонисты каннабиноидных рецепторов и растущее в настоящее время семейство агонистов пептидных гормонов желудочно-кишечного тракта.

Несмотря на разнообразие химического строения, действие препаратов, когда-либо используемых для лечения ожирения, можно разделить на 3 направления: снижение потребления энергии, увеличение расхода энергии и уменьшение абсорбции питательных веществ.

## Гормоны щитовидной железы

Гормоны щитовидной железы были первым лекарственным агентом, использованным для лечения ожирения, возможно, из-за ошибочного представления о том, что чрезмерное отложение жира в организме является проявлением «скрытого» гипотиреоза [13]. Хотя экзогенные тироксин и трийодтиронин вызывали быструю потерю веса, до 80% потерянной массы приходилось на безжировую ткань. Длительный прием тиреоидных гормонов в супрафизилогических дозах, необходимых для снижения массы тела, был неизбежно ассоциирован с развитием артифициального тиреотоксикоза, наиболее серьезными последствиями которого являются тахикардия, нарушения ритма сердца, сердечная недостаточность, остеопороз. Ввиду этих осложнений представляется удивительным, что тироксин широко использовался для лечения ожирения вплоть до 1980-х годов [14].

## Динитрофенол

Динитрофенол является респираторным ядом, разобщающим дыхание и фосфорилирование и увеличивающим скорость метаболизма. Динитрофенол был введен для лечения ожирения в 1933 году и назначался либо в качестве монотерапии, либо в сочетании с гормоном щитовидной железы [14]. Динитрофенол вызывал быструю потерю веса, сопровождающуюся ощущением жара, потливостью или развитием лихорадки. По оценкам, этим препаратом могли лечиться до 100 000 человек. Тяжелая токсичность динитрофенола, включающая дерматит, агранулоцитоз, гепатотоксичность, нарушение зрения и смерть, привели к прекращению его использования. В 1938 году препарат был снят с производства [15].

## Амфетамины

Амфетамины воздействуют на рецепторы гипоталамуса, высвобождая норадреналин и, в меньшей степени, дофамин и серотонин, повышая активность центральной нервной системы (ЦНС) и расход энергии в состоянии покоя, а также снижая аппетит и потребление пищи, что приводит к снижению веса [15]. Комбинация амфетамина с гормоном щитовидной железы, наперстянкой и диуретиками, назначалась с 1940-х по 1960-е годы и была известна как «радужные таблетки». Однако такой подход к лечению ожирения вызывал привыкание, артериальную гипертензию, тяжелую миокардиальную токсичность и внезапную смерть [7].

Аминорекс — амфетаминоподобный симпатомиметический препарат с анорексическими свойствами, был представлен в Европе в 1965 году для лечения ожирения. Эпидемия хронической легочной гипертензии, вызванная прекапиллярной сосудистой обструкцией, среди принимавших аминорекс была отмечена в 1967 г. и сохранялась до 1972 г. Смертность среди лиц, принимавших аминорекс, составила 50%. Препарат был изъят из продаж в 1968 г. [16].

Фенфлурамин является препаратом, который одновременно стимулирует высвобождение и ингибирует пресинаптический обратный захват серотонина без симпатомиметической или ЦНС-стимулирующей активности. Комбинация фенфлурамина с симпатомиметиком фентермином была представлена в 1992 г. как эффективная долгосрочная терапия ожирения, не имеющая серьезных побочных эффектов [17]. Хотя подход «фен-фен» был полезен для демонстрации того, что длительный прием лекарств необходим для эффективного лечения ожирения, проблемы с этими препаратами стали очевидны уже в 1996 г. В том же году группа случаев первичной легочной гипертензии была соотнесена с использованием производных фенфлурамина в Западной Европе [18]. В следующем году аналогичный случай был зарегистрирован у пациента в Соединенных Штатах, который принимал фенфлурамин и фентермин всего 23 дня [18]. Это повторение опыта приема аминорекса вполне объяснимо, учитывая структурное сходство этих анорексигенных препаратов. Еще большую озабоченность вызвал отчет 1997 года, связывающий использование фенфлурамина с формированием порока сердца [19].

В 1996 г. в США после 52-недельного испытания был одобрен препарат дексфенфлурамин, изомер фенфлурамина. Использование дексфенфлурамина стало широко распространенным, и после первого года его утверждения еженедельно выписывалось до 85 000 рецептов. Однако спустя 6 месяцев серия случаев дегенерации митрального клапана у пациентов, принимавших дексфенфлурамин, привела к ограничению его использования.

Поражения митрального и аортального клапанов (вальвулопатия), вызванные фенфлурамином, напоминали клапанные аномалии, наблюдаемые при карциноидном синдроме, другом состоянии избытка серотонина. Ранняя серия исследований Управления по санитарному надзору за качеством пищевых продуктов и медикаментов (FDA) выявила аномальные эхокардиограммы у 92 из 291 пациента (32%), получавших фенфлурамин или дексфенфлурамин в срок до 24 месяцев [20]. Однако последующие исследования пациентов, которым была выполнена эхокардиография как до, так и после приема данных препаратов, показали, что частота новых случаев клапанной регургитации составляет 4,3–16,5%, большинство из которых были бессимптомными [21]. Было обнаружено, что частота клапанных поражений у пациентов, получавших лечение менее 3 месяцев, составляет менее 3% [22]. Эхокардиограммы, полученные у пациентов с поражением клапанов через 6–24 месяца после прекращения терапии фенфлурамином или дексфенфлурамином, обычно демонстрировали стабильность или уменьшение регургитации [23].

Тем не менее, учитывая данные о вальвулопатиях, а также большое количество судебных исков, последовавших вслед за использованием данных препаратов, в сентябре 1997 года производитель добровольно вывел с рынка как фенфлурамин, так и дексфенфлурамин.

Фенилпропаноламин — симпатомиметический препарат, содержащийся в многочисленных средствах для подавления аппетита и средствах от кашля или простуды, является еще одним амфетаминоподобным препаратом для контроля массы тела, который в 2000 г. был удален с рынка из-за его связи с повышением частоты геморрагических инсультов. Исследование 702 мужчин и женщин в возрасте 18–49 лет показало отношение шансов 16,58 для связи фенилпропаноламин-содержащих средств, подавляющих аппетит, и геморрагического инсульта у женщин [24].

## Сибутрамин

Сибутрамин, производное амфетамина, был одобрен для лечения ожирения в США и Канаде в 1997 г., а в Европе — в 1999 г. Сибутрамин ингибирует обратный нейрональный захват серотонина и норадреналина в центральной нервной системе, тем самым усиливая чувство сытости, снижая аппетит и увеличивая термогенез [25].

Эффективность сибутрамина (Meridia, Abbott Laboratories) изучалась в течение нескольких лет в ходе 46 клинических испытаний с участием 9303 пациентов с избыточной массой тела или ожирением, 5812 (62%) из которых получали препарат. В исследованиях сообщалось о стойкой потере массы тела со средним снижением, за вычетом плацебо, 4,2 кг в течение 12 месяцев [26]. Нежелательные явления включали повышение систолического и диастолического артериального давления и частоты сердечных сокращений даже у пациентов с исходно нормальным артериальным давлением [26]. Сибутрамин был одобрен в 1997 г. с учетом противопоказаний к его применению у пациентов с ишемической болезнью сердца, инсультом, застойной сердечной недостаточностью, аритмией или неконтролируемой артериальной гипертензией. Опасения по поводу безопасности сибутрамина после его лицензирования впервые были высказаны в Бельгии в 1999 году, когда у значительного числа пациентов были зарегистрированы повышенное артериальное давление и частота сердечных сокращений. В результате этого европейский Комитет по лекарственным средствам для человека (Committee for Medicinal Products for Human Use (CHMP)), принадлежащий Европейскому агентству по оценке лекарственных средств (EMA), рассмотрел безопасность и эффективность сибутрамина и пришел к выводу, что регистрационное удостоверение сибутрамина может быть сохранено при условии проведения дополнительного клинического исследования, направленного на оценку его влияния на факторы сердечно-сосудистого риска. В 2002 г. в Италии было зарегистрировано необычное количество неблагоприятных сердечно-сосудистых событий при приеме сибутрамина, что привело к временной приостановке его применения в этой стране, после чего было инициировано крупное официальное европейское исследование для полной оценки профиля сердечно-сосудистой безопасности сибутрамина. В продолжение этой оценки безопасности в 2005 году было начато 5-летнее исследование SCOUT, в котором 9804 субъекта были рандомизированы для получения сибутрамина или плацебо на период более 3 лет. В отличие от предыдущих исследований фазы II и III, в исследование SCOUT специально включали лиц с сердечно-сосудистыми заболеваниями (ССЗ), высоким риском сердечно-сосудистых событий, высоким индексом массы тела (ИМТ), церебральными сосудистыми заболеваниями, заболеваниями периферических артерий или СД2 с хотя бы одним дополнительным сердечно-сосудистым фактором риска, чтобы увеличить количество нежелательных сердечно-сосудистых явлений [27]. Тем не менее ожидаемая частота сердечно-сосудистых событий (ожидаемая ~7%) не была достигнута, в связи с чем исследование было расширено и ограничено только тремя группами: пациенты только с СД 2, пациенты только с ССЗ и пациенты с СД2 и ССЗ (группа самого высокого риска).

Было обнаружено, что у пациентов, получавших сибутрамин, отмечалось значимое повышение относительного риска (ОР) сердечно-сосудистых событий (1,16 [ 95% доверительный интервал (ДИ): 1,03–1,31; p=0,02]), однако повышенный риск сердечно-сосудистых событий существенно не отличался, когда указанные три группы субъектов рассматривались по-отдельности [ только СД2 — 1,01 (95% ДИ: 0,74–1,38), только ССЗ — 1,28 (95% ДИ: 0,92–1,78), СД2 с ССЗ — 1,18 (95% ДИ: 1,02–1,37)] [27]. Тем не менее из-за общего потенциального риска, связанного с этим препаратом, FDA рекомендовало добровольно отозвать сибутрамин с рынков США и Канады, что было выполнено Abbott Laboratories в октябре 2010 г.

Еще до того, как были опубликованы полные результаты исследования SCOUT, продолжающиеся опасения по поводу безопасности сибутрамина в Европе привели к тому, что CHMP рекомендовал отозвать сибутрамин, и с января 2010 г. его использование в Европе было приостановлено.

Повторный анализ данных SCOUT показал, что сибутрамин значительно снижает риск сердечно-сосудистых осложнений и смертности у пациентов, снизивших массу тела; повышенный сердечно-сосудистый риск наблюдался только у пациентов с высоким риском, которые не похудели, но продолжали принимать препарат только для выполнения протокола исследования [28].

В Российской Федерации (РФ) препараты, содержащие сибутрамин, не отозваны и имеют активную регистрацию. С января 2008 г. сибутрамин входит в утвержденный правительством список сильнодействующих препаратов, то есть отпускается только по рецепту (форма 107-1/у) [29]. Сибутрамин может применяться у взрослых моложе 65 лет с учетом противопоказаний (ишемическая болезнь сердца, декомпенсированная хроническая сердечная недостаточность, врожденные пороки сердца, окклюзивные заболевания периферических артерий, тахикардия, нарушения ритма сердца, цереброваскулярные заболевания, неконтролируемая артериальная гипертензия). Дополнительным фактором, лимитирующим эффективное использование сибутрамина в лечении ожирения, является ограничение максимальной длительности его применения сроком 1 год.

## Орлистат

Препараты для лечения ожирения, не содержащие амфетамин, стали разрабатываться к концу 1990-х годов. Первым на рынок вышел орлистат (Roche, Швейцария), одобренный в Европе в 1998 г. и США в 1999 г. и доступный по сей день, в том числе на территории России. Орлистат ингибирует панкреатическую липазу, таким образом примерно на 30% уменьшая расщепление и всасывание жиров в тонком кишечнике. Клинические испытания орлистата (например, XENDOS) продемонстрировали значимо большую потерю веса по сравнению с плацебо [ 5,8 кг против 3,0 кг; р<0,001] [30]; однако метаанализ 15 исследований с орлистатом показал, что при лечении до 4 лет общее среднее снижение массы тела с поправкой на плацебо составило всего около 2,9 кг (2,9%) [26]. При применении орлистата возникла только одна значимая проблема безопасности: его потенциальная гепатотоксичность. Однако недавний метаанализ применения орлистата в Великобритании не показал повышенного риска гепатотоксичности [31]. Менее опасные побочные эффекты со стороны желудочно-кишечного тракта, такие как боль, вздутие и дискомфорт в животе, маслянистый или жидкий стул и императивные позывы к дефекации, встречаются очень часто, что, к сожалению, ограничивает долгосрочную переносимость орлистата.

## Римонабант

Римонабант был разработан и одобрен в Европе в 2006 г. (Sanofi, Франция). Данный препарат является антагонистом каннабиноидных рецепторов CB1, тем самым снижая аппетит [32]. Нежелательные явления, зарегистрированные во время клинических испытаний римонабанта, включали повышенную частоту психических расстройств, включая депрессию, тревогу и суицидальные мысли [33]. Великобритания была первой страной, где продавался римонабант, и к 2008 году препарат был доступен в 56 странах. В России, США, Канаде препарат зарегистрирован не был. В октябре 2008 г. в заявлении EMA рекомендовалось приостановить действие всех разрешений на продажу римонабанта в связи с теми же рисками психических расстройств, что привело к его полному отзыву в Европе и в итоге во всем мире.

## НОВЫЕ И ГРЯДУЩИЕ МЕТОДЫ ЛЕЧЕНИЯ ОЖИРЕНИЯ

За последние несколько десятилетий стало совершенно ясно, что ожирение характеризуется сложными патофизиологическими механизмами, определяющими его хроническое, самоподдерживающееся и рецидивирующее течение. Кроме того, новая информация о нейронных цепях, контролирующих прием пищи, и их гормональной регуляции расширила наше понимание энергетического гомеостаза. Каждый новый сигнальный путь, обнаруженный в гипоталамусе, является потенциальной мишенью для разработки лекарств для лечения ожирения. Однако, поскольку эти центральные пути задействованы во множестве функций, неудивительно, что некоторые фармакологические агенты в итоге потерпели неудачу из-за неожиданных побочных эффектов, которые могут быть выявлены только во время клинических испытаний, а иногда и через много лет после регистрации препарата. Тем не менее, несмотря на многие предыдущие неудачи в процессе разработки лекарств от ожирения, производители в последние два десятилетия прикладывали настойчивые усилия для разработки новых, более безопасных и более переносимых препаратов. В течение этого времени появилось несколько новых потенциально эффективных и безопасных фармакотерапевтических средств для долгосрочного снижения массы тела. Кроме того, стало понятным, что идеальный препарат для лечения ожирения должен приводить к значимой и устойчивой потере веса, одновременно снижая риск сердечно-сосудистых событий и других сопутствующих заболеваний, не вызывать зависимость и тахифилаксию, а также не иметь побочных эффектов, значительно ограничивающих его использование.

## Лоркасерин

Первым из них является лоркасерин, одобренный в США в 2012 г. (Arena Pharmaceuticals, США). Лоркасерин действует на рецепторы 5-гидрокситриптамина 2C (5-HT2C), вызывая высвобождение и ингибируя последующий обратный нейрональный захват серотонина (5-HT). Из-за его низкой специфичности в отношении 5-HT2B-рецептора (примерно в 100 раз ниже, чем у 5-HT2C-рецептора) лоркасерин имеет низкий риск возникновения вальвулопатии при длительном применении [34]. В двух 52-недельных исследованиях III фазы сообщалось, что у пациентов со средней исходной массой тела ~100 кг терапия лоркасерином привела к потере массы тела до 6% (по сравнению с 2–3% при применении плацебо; p=0,001). Частота нежелательных явлений и прекращение лечения из-за них в обоих исследованиях в целом были низкими (6–7%), при этом наиболее частым побочным эффектом были головные боли [35]; однако в целом около трети пациентов выбыли из первого исследования, а во втором доля выбывших выросла до более чем 40%. Из-за предшествующего высокого риска развития вальвулопатии при применении других агонистов серотониновых рецепторов (например, фенфлурамина) всем участникам исследования III фазы были выполнены эхокардиограммы, что привело к окончательному заключению, что лоркасерин не вызывает фиброза ткани клапанов сердца. Хотя лоркасерин хорошо переносится, долгосрочных исследований его сердечно-сосудистой безопасности не проводилось.

В Европе была подана заявка на одобрение лоркасерина; тем не менее заявка была отозвана в мае 2013 г. после того, как EMA заявило, что польза лоркасерина в отношении снижения массы тела не перевешивает его риски, которые включали возможность увеличения частоты психических расстройств и вальвулопатии, которые наблюдались у некоторых пациентов во время испытаний фазы III. В Российской Федерации лоркасерин также не был зарегистрирован.

В 2020 году FDA потребовало отозвать лоркасерин из-за данных наблюдательных исследований, показавших возможное повышение онкологической заболеваемости на фоне его приема.

## Фентермин/топирамат пролонгированного действия

Комбинированная терапия фентермином и топираматом пролонгированного высвобождения (extended‐release (ER)) была разработана компанией Vivus (США). Хотя точный механизм действия остается неизвестным, считается, что фентермин опосредует высвобождение катехоламинов (включая норадреналин и дофамин) в гипоталамусе, в то время как топирамат усиливает активность нейротрансмиттера гамма-аминобутирата, модулирует потенциалзависимые ионные каналы и ингибирует АМРА (α-амино-3-гидрокси-5-метил-4-изоксазолпропионовая кислота)/каинит, возбуждающие глутаматные рецепторы и карбоангидразу [7]. Хотя первоначально препарат был отклонен из-за опасений по поводу повышенного риска депрессии и когнитивных расстройств, а также сердечно-сосудистых осложнений, решение было пересмотрено, и фентермин/топирамат ER одобрен FDA для снижения массы тела в 2012 году. Ключевые клинические испытания, которые сыграли важную роль в его одобрении, включают EQUATE, CONQUER, EQUIP и SEQUEL, в которых был продемонстрирован значимый дозозависимый эффект фентермина/топирамата ER на снижение массы тела [36–39]. Так, в 56-недельном исследовании CONQUER терапия максимальной дозой препарата (фентермин 15,0 мг плюс топирамат 92,0 мг) привела к снижению массы тела на 12,4% по сравнению с 1,6% в группе плацебо. Также наблюдались улучшения со стороны показателей сердечно-сосудистой системы [37]. Исследование SEQUEL, представляющее собой расширенное на 2 года исследование CONQUER, подтвердило последовательность и надежность результатов, кроме того, в группе с максимальной дозой наблюдалось снижение частоты впервые выявленного СД2 на 76% [39].

Наиболее частыми побочными реакциями на фентермин/топирамат ER в этих исследованиях были парестезия, головокружение, дисгевзия, бессонница, запор и сухость во рту без повышения риска тяжелых сердечно-сосудистых событий. Препарат противопоказан при беременности из-за связанного с топираматом повышенного риска орофациальных расщелин [40]. В Европе разрешение на продажу фентермина/топирамата ER было отклонено EMA как в 2012-м, так и в 2013 году из-за соображений безопасности в отношении долгосрочного воздействия на сердце и кровеносные сосуды, а также психиатрических и когнитивных эффектов. В РФ фентермин/топирамат ER не зарегистрирован.

## Налтрексон/бупропион

Налтрексон/бупропион был разработан компанией Orexigen Therapeutics (США). Хотя бупропион является относительно слабым ингибитором захвата норэпинефрина и дофамина, он стимулирует гипоталамические проопиомеланокортиновые (POMC) нейроны, что приводит к активации меланокортиновых рецепторов и индукции снижения массы тела за счет подавления аппетита и увеличения расхода энергии. Налтрексон действует как антагонист опиоидных рецепторов, которые обычно индуцируют опосредованное отрицательной обратной связью подавление активации РОМС, и, таким образом, действует синергетически, пролонгируя действие бупропиона на метаболизм.

Первоначально эффективность налтрексона/бупропиона оценивалась в трех исследованиях фазы III у пациентов без СД2, которые продемонстрировали снижение массы тела от 6,1 до 9,3% по сравнению с исходной, и одного исследования фазы III у пациентов с СД2, в котором снижение массы тела составило до 5% [7][41][42]. На основании этих данных, а также промежуточных данных исследования сердечно-сосудистой безопасности налтрексона/бупропиона (исследование LIGHT), FDA одобрило налтрексон/бупропион в сентябре 2014 г. с требованием проведения постмаркетинговых исследований сердечно-сосудистого риска [7]. Исследование LIGHT было прекращено досрочно после того, как спонсор публично обнародовал конфиденциальные благоприятные промежуточные результаты, когда произошло только 25% ожидаемых сосудистых событий, что затруднило интерпретацию сердечно-сосудистой безопасности этого комбинированного препарата. В странах Евросоюза препарат одобрен Европейским агентством по оценке лекарственных средств (EMA) для лечения ожирения в 2015 г. В Российской Федерации налтрексон/бупропион не зарегистрирован. В недавнем метаанализе 35 рандомизированных клинических испытаний было показано отсутствие повышенного риска сердечно-сосудистых событий или комбинированной конечной точки (MACE) на фоне применения налтрексона/бупропиона [43]. Наиболее частыми побочными эффектами препарата являются тошнота, головная боль, запор, головокружение, рвота, бессонница и сухость во рту, при этом повышенного риска депрессии или суицидальных мыслей не зарегистрировано [44].

## Агонисты рецепторов гормонов желудочно-кишечного тракта (инкретинов)

Изучение пептидных гормонов желудочно-кишечного тракта и понимание механизмов их влияния на энергетический гомеостаз, пищевое поведение и массу тела положило начало новой эры в медикаментозном лечении ожирения. Гормоны желудочно-кишечного тракта, участвующие в регуляции пищевого поведения, секретируются либо в ответ на прием пищи (холецистокинин (CCK), пептид тирозин-тирозин (PYY), глюкагоноподобный пептид 1 (GLP-1), глюкозозависимый инсулинотропный полипептид (GIP), оксинтомодулин), либо в ответ на депривацию питательных веществ (грелин, глюкагон, фактор роста фибробластов 21 (FGF21)). Помимо гомеостатической регуляции потребления пищи, голод и насыщение подвержены влиянию внешних факторов, таких как вид, запах и вкус пищи, что связано с эндогенной системой вознаграждения и формирует гедонистическое пищевое поведение. Области мозга, участвующие в гедонистическом пищевом поведении, включают области рядом с гипоталамусом и стволом мозга, дофаминергические центры вознаграждения в мезолимбической области мозга, а также гиппокамп и кору [45].

Дугообразное ядро гипоталамуса представляет собой ключевой узел контроля гомеостатического потребления пищи, который включает орексигенные нейроны, экспрессирующие нейропептид Y (NPY) и агути-подобный пептид (AgRP), и анорексигенные нейроны, экспрессирующие проопиомеланокортин (POMC) и кокаин-амфетамин-регулируемый транскрипт (CART). Активация нейронов NPY/AgRP приводит к секреции AgRP, который стимулирует прием пищи посредством блокирования рецептора меланокортина 4 (MC4R), тогда как активация нейронов POMC/CART приводит к секреции α-меланоцитостимулирующего гормона (α-MSH), который активирует MC4R и подавляет прием пищи [45].

## Лираглутид

Лираглутид был первым агонистом рецепторов GLP-1, одобренным для лечения ожирения. В дозах 1,2 мг и 1,8 мг он первоначально был разработан компанией Novo Nordisk (Дания) для лечения СД2. Молекула лираглутида имеет период полувыведения 13 часов, что значительно продлевает активацию рецептора GLP-1 (период полувыведения нативного GLP-1 составляет ~1,5 мин), тем самым усиливая глюкозозависимую стимуляцию секреции инсулина и оказывая выраженный глюкозозависимый гипогликемический эффект. По мере клинического использования лираглутида в качестве сахароснижающего препарата у пациентов с СД2 стало очевидным его значимое влияние на подавление аппетита и снижение массы тела. Выяснилось, что эндогенный GLP-1 снижает потребление пищи посредством центральных механизмов, которые, по-видимому, включают прямую активацию анорексигенных нейронов POMC/CART, а также подавление орексигенных нейронов NPY/AgRP [46]. Агонисты рецепторов GLP-1, по всей видимости, также модулируют гедонистическое потребление пищи, воздействуя на дофаминергическую систему вознаграждения мозга и уменьшая удовольствие, получаемое от приема пищи [47].

Дополнительным механизмом, обуславливающим снижение массы тела под действием агонистов рецепторов GLP-1, является подавление моторики желудка, что ведет к замедлению эвакуации пищи и усилению чувства «наполненности».

Действительно, 5-недельное неполное перекрестное исследование лираглутида показало, что он индуцирует снижение массы тела, скорее всего, за счет снижения аппетита и уменьшения потребления пищи [48]. Эти данные побудили исследовать применение более высокой дозы лираглутида (3,0 мг в день) в клинических испытаниях фазы III (программа SCALE) для изучения безопасности и эффективности данной терапии для контроля массы тела у пациентов с ожирением, как с СД2, так и без него. В крупнейшем исследовании программы SCALE, Obesity and Prediabetes, изучались эффекты лираглутида в дозе 3,0 мг у 3731 человека с ИМТ≥30 кг/м² или ≥27 кг/м² с дополнительными сопутствующими заболеваниями, с преддиабетом или без него, в течение 56 недель [49]. На 56-й неделе участники, получавшие лираглутид 3,0 мг, потеряли 8,0% (8,4 кг) массы тела по сравнению с 2,6% (2,8 кг) в группе плацебо [ оценочная разница в лечении -5,4%, (5,6 кг), p<0,001]. Помимо снижения массы тела, наблюдались и другие преимущества, в том числе улучшение гликемического контроля, артериального давления и уровня липидов. Лираглутид в дозе 3,0 мг в целом хорошо переносился, при этом наиболее частыми нежелательными явлениями были тошнота и диарея в течение первых 4–8 недель лечения [49].

На основании программы клинических испытаний III фазы SCALE лираглутид в дозе 3,0 мг был зарегистрирован для лечения ожирения в большинстве стран мира: в США — в 2014 г., в странах Евросоюза — в 2015 г., в Российской Федерации — в 2016 г.

## Семаглутид

Семаглутид — новейший агонист рецепторов GLP-1, одобренный FDA и ЕМА для длительного лечения ожирения в 2021 году. Как и лираглутид, первоначально он был разработан компанией Novo Nordisk для лечения СД2. Одобрение семаглутида 2,4 мг в неделю для снижения массы тела было основано на результатах программы исследований STEP (1–8). В исследовании STEP 1 в общей сложности 1961 взрослый с ожирением или избыточной массой тела и сопутствующими коморбидными заболеваниями, за исключением СД, были рандомизированы для получения семаглутида 2,4 мг еженедельно или плацебо, в дополнение к модификации образа жизни [50]. Исследование показало, что среднее снижение массы тела от исходного уровня до 68-й недели составило -14,9% в группе семаглутида и -2,4% в группе плацебо при предполагаемой разнице в лечении -12,4% (p<0,001). По сравнению с плацебо у большего числа участников в группе семаглутида было достигнуто снижение массы тела на ≥5% (86,4 против 31,5%), ≥10% (69,1 против 12,0%), ≥15% (50,5 против 4,9%) и ≥20% (32,0 против 1,7%). Среди участников с исходным преддиабетом 84% вернулись к нормогликемии в группе вмешательства по сравнению с 38% в контрольной группе.

Исследование STEP 4 продемонстрировало положительный эффект длительного лечения семаглутидом для снижения массы тела. Испытание включало 20-недельный вводный период, в течение которого все участники испытания получали семаглутид 2,4 мг еженедельно, после чего следовал 48-недельный период, когда группа вмешательства продолжала прием семаглутида 2,4 мг еженедельно, а контрольная группа получала плацебо. Группа семаглутида продолжала снижать, а затем стабилизировала массу тела в течение следующих 48 недель, тогда как в группе плацебо отмечались набор массы тела и ухудшение сердечно-сосудистых факторов риска [51].

Пероральная форма семаглутида, которая в настоящее время зарегистрирована для лечения СД2, также представляется перспективной в рамках фармакотерапии ожирения. Так, в исследовании 3 фазы OASIS 1 пероральный семаглутид в дозе 50 мг ежедневно продемонстрировал эффективность, сравнимую с таковой для подкожного введения семаглутида 2,4 мг в неделю: среднее снижение массы тела к 68-й неделе исследования составило -15,1% по сравнению с -2,4% при приеме плацебо [52].

В Российской Федерации семаглутид в инъекционной и пероральных формах зарегистрирован для лечения СД2.

Среди нежелательных явлений терапии семаглутидом, как и у других агонистов GLP-1, наиболее часто встречаются желудочно-кишечные симптомы: тошнота, констипация, диарея (74,2 против 47,9% в группе плацебо). Серьезные, но менее распространенные побочные эффекты включают заболевание желчного пузыря (2,6%) и панкреатит (0,2%). Различий в частоте возникновения доброкачественных и злокачественных новообразований между группами семаглутида и плацебо выявлено не было.

## Полиагонисты инкретинов

Одновременно со структурной оптимизацией селективных моноагонистов рецепторов GLP-1 проводились исследования по фармакологическому использованию того факта, что организмы млекопитающих регулируют энергетический баланс посредством гораздо большего количества гормонов. Наиболее заметным прорывом в этом направлении стало открытие полиагонистов, которые одновременно нацелены на рецепторы GLP-1, GIP и/или глюкагона.

В начале 2016 г. компания Eli Lilly and Company (США) разработала новый сахароснижающий препарат тирзепатид [53]. Тирзепатид представляет собой синтетически полученную пептидную молекулу, которая действует в качестве агониста рецепторов как GIP, так и GLP-1. Из-за этого уникального свойства двойной активности его также называют «твинкретином». Период полувыведения препарата составляет около 5 дней, таким образом, достаточно его подкожного введения один раз в неделю.

Эффективность и безопасность тирзепатида в качестве гипогликемизирующего препарата у пациентов с СД2 была продемонстрирована в серии исследований SURPASS. В 2022 г. тирзепатид был одобрен в качестве сахароснижающего препарата для лечения СД2 в США, странах Евросоюза, Канаде, Австралии, ОАЭ.

В исследованиях SURPASS 1–5 терапия тирзепатидом сопровождалась значительным снижением массы тела, окружности талии и улучшением метаболического профиля пациентов с СД2, что послужила поводом для изучения эффективности данного препарата для лечения пациентов с ожирением без СД2. С этой целью было проведено исследование SURMOUNT-1, двойное слепое рандомизированное контролируемое клиническое исследование фазы III, которое показало, что у участников с ожирением без СД2 тирзепатид в дозе 5 мг, 10 мг или 15 мг один раз в неделю в течение 72 недель приводил к существенному и устойчивому снижению массы тела [54]. Тирзепатид улучшал кардиометаболические показатели, такие как окружность талии. Среднее снижение массы тела по истечении 72 недель терапии составило15,0%, -19,5% и -20,9% при применении тирзепатида в дозе 5 мг, 10 мг и 15 мг соответственно, по сравнению с -3,1% в группе плацебо. Доля участников со снижением массы тела на 5% и более составила 85%, 89% и 91% при приеме тирзепатида 5 мг, 10 мг и 15 мг соответственно, и 35% при приеме плацебо; 50% участников группы 10 мг и 57% участников группы 15 мг снизили массу тела на 20% и более по сравнению с 3% в группе плацебо. Примечательно, что в отличие от предшественников (лираглутид, семаглутид) терапия тирзепатидом сопровождалась продолжающимся снижением массы тела в течение всего периода испытания (72 недели) без выхода на «плато» [54]. В ноябре 2023 г. тирзепатид получил одобрение FDA в качестве препарата для лечения ожирения.

Профиль безопасности тирзепатида в целом аналогичен другим инкретиновым препаратам для лечения ожирения. Наиболее частыми побочными эффектами тирзепатида, проявляющимися в большинстве случаев, являются транзиторные желудочно-кишечные явления легкой и средней степени тяжести (тошнота, диарея, запор), возникающие в основном в период повышения дозы. Терапия тирзепатидом также сопровождалась повышением частоты холецистита по сравнению с плацебо, что, по всей видимости, обусловлено быстрым снижением массы тела и наблюдается также при других эффективных методах лечения ожирения, таких как бариатрическая хирургия и лечение агонистами рецептора GLP-1.

## ПЕРСПЕКТИВЫ ФАРМАКОТЕРАПИИ ОЖИРЕНИЯ

Появление и успешное применение семаглутида и тирзепатида привели к быстрому расширению спектра препаратов, разрабатываемых для контроля массы тела. В настоящее время изучается несколько потенциально высокоэффективных терапевтических средств на основе гормонов, регулирующих нейроэндокринные механизмы контроля приема пищи, жировой массы тела и гомеостаза энергии. Большинство новых препаратов являются агонистами рецепторов этих пептидных гормонов: глюкагоноподобного пептида 1 (GLP-1), глюкозозависимого инсулинотропного полипептида (GIP), глюкагона, амилина, оксинтомодулина и пептида YY, — влияющих, по-видимому, на регуляцию жировой массы тела и гомеостаза энергии. Некоторые агонисты рецепторов GLP-1-глюкагона и GIP-GLP-1-глюкагона в настоящее время находятся в стадии клинической разработки, учитывая предположение, что включение агонизма рецепторов глюкагона может дополнительно снизить потребление энергии, увеличить расход энергии или и то и другое, таким образом, потенциально повышая эффективность. Примером может служить сурводутид — двойной агонист рецепторов GLP-1 и глюкагона. В исследовании 2 фазы с участием 386 пациентов с ожирением терапия сурводутидом в течение 46 недель приводила к снижению массы тела вплоть до 14,9% от исходной [55]. В настоящее время сурводутид проходит 3 фазу исследований.

Также интерес представляет орфоглипрон — первый непептидный агонист рецепторов GLP-1 для ежедневного перорального приема. В исследовании 2 фазы с участием 272 лиц с избыточной массой тела или ожирением среднее изменение массы тела к 36-й неделе терапии варьировалось от -9,4% до -14,7% в группе орфорглипрона и составило -2,3% в группе плацебо. Снижение массы тела как минимум на 10% к 36-й неделе произошло у 46–75% участников, получавших орфорглипрон, по сравнению с 9%, получавших плацебо [56].

Еще одной перспективой в лекарственном лечения ожирения является использование комбинации нескольких пептидных агонистов. Так, высокую эффективность в клинических испытаниях показал препарат КагриСема, представляющий собой комбинацию семаглутида с агонистом амилина кагрилинтидом. В рандомизированном клиническом исследовании 2 фазы 92 пациента с СД2 и ожирением были рандомизированы на прием КагриСема (n=31), семаглутида (n=31) или кагрилинтида (n=30). На 32-й неделе исследования среднее снижение уровня гликированного гемоглобина составило -2,2%, -1,8% и -0,9% в группах КагриСема, семаглутида и кагрилинтида соответственно, среднее снижение массы тела — -15,6%, -5,1% и -8,1% в соответствующих группах [57].

Большие надежды возлагаются на ретатрутид, который является первым тройным агонистом пептидных гормонов кишечника, разработанным для лечения СД2 и ожирения компанией Eli Lilly and Company (США). Ретатрутид представляет собой пептид, проявляющий агонизм к рецепторам GIP, GLP-1 и глюкагона. По сравнению с лигандами эндогенных рецепторов, ретатрутид менее активен в отношении рецепторов глюкагона и GLP-1 человека (в 0,3 и 0,4 раза соответственно) и более активен в отношении рецептора GIP человека (в 8,9 раза) [58]. Фармакокинетика ретатрутида считается дозозависимой; период его полураспада составляет примерно 6 дней, таким образом, его можно применять еженедельно. В исследовании фазы 1b с участием пациентов с диабетом 2 типа лечение ретатрутидом привело к снижению массы тела примерно на 10% в группе с самой высокой дозой (12 мг) через 12 недель [59].

В июне 2023 года были опубликованы результаты испытания 2 фазы, посвященного изучению эффективности и безопасности ретатрутида в различных дозах у лиц с ожирением без СД2 [60]. В исследование было включено 338 взрослых с ожирением. По истечении 48 недель лечения среднее снижение массы тела в группах ретатрутида составило -8,7% в группе 1 мг, -17,1% в комбинированной группе 4 мг, -22,8% в комбинированной группе 8 мг и -24,2% в группе 12 мг в неделю по сравнению с -2,1% в группе плацебо. Снижение массы тела на ≥5%, ≥10% и ≥15% наблюдалось, соответственно, у 92%, 75% и 60% участников, принимавших 4 мг ретатрутида; 100%, 91% и 75% тех, кто получил 8 мг; 100%, 93% и 83% тех, кто получил 12 мг; и 27%, 9%, и 2% тех, кто получил плацебо. Наиболее частыми нежелательными явлениями в группах ретатрутида были желудочно-кишечные симптомы, которые были дозозависимыми, в основном — легкой или умеренной тяжести и частично смягчались при более низкой начальной дозе (2 мг против 4 мг). Таким образом, впервые для медикаментозной терапии ожирения эффективность препарата оказалась сравнима с эффектом бариатрической хирургии.

## ЗАКЛЮЧЕНИЕ

В течение последнего столетия человечество столкнулось с нарастающей эпидемией ожирения и коморбидных заболеваний, обусловившей необходимость разработки эффективных и безопасных методов контроля массы тела. История медикаментозной терапии ожирения длится немногим более 100 лет, однако характеризуется множеством взлетов и падений. Понимание природы ожирения как хронического заболевания, а также изучение молекулярных механизмов контроля пищевого поведения и энергетического гомеостаза послужило основой для создания новых эффективных лекарственных препаратов для снижения массы тела. Возможно, мы стоим на пороге новой эры в медикаментозном лечении ожирения, однако многие проблемы, такие как доступность, переносимость и возможность длительного применения фармакотерапии ожирения еще предстоит решить.

## ДОПОЛНИТЕЛЬНАЯ ИНФОРМАЦИЯ

Источники финансирования. Работа выполнена по инициативе авторов без привлечения финансирования.

Конфликт интересов. Авторы декларируют отсутствие явных и потенциальных конфликтов интересов, связанных с содержанием настоящей статьи.

Участие авторов. Все авторы одобрили финальную версию статьи перед публикацией, выразили согласие нести ответственность за все аспекты работы, подразумевающую надлежащее изучение и решение вопросов, связанных с точностью или добросовестностью любой части работы.
